# The NFAT5 chronicles: a transcription factor’s tale of hypoxia, pulmonary drama, and endothelial resilience to hypoxia

**DOI:** 10.1007/s00424-025-03080-w

**Published:** 2025-03-28

**Authors:** Dörthe M. Katschinski

**Affiliations:** Institute of Cardiovascular Physiology, University Medical Center, Humboldtallee 23, 37073 Göttingen, Germany

In the ever-evolving world of cardiovascular research, where molecular players engage in an intricate dance to maintain physiological harmony, a new protagonist has emerged: nuclear factor of activated T-cells 5 (NFAT5). This unsung hero has long been overlooked in the grand narrative of endothelial cell adaptation to stress, yet a recent study has given it a spotlight [2]. The pulmonary endothelium is a battleground where hypoxia forces cells into survival mode [3]. A sophisticated transcriptional program is activated, spearheaded by familiar molecular sentinels like the hypoxia inducible factor (HIF)−1α [1]. However, the study reveals that NFAT5, previously best known for its osmoregulatory roles, is indispensable in orchestrating endothelial responses to hypoxia. When NFAT5 is deleted from endothelial cells, the results are nothing short of a physiological soap opera: rampant vascular fibrosis, unchecked smooth muscle proliferation, and an ominous rise in pulmonary artery pressure leading to right ventricular dysfunction.

## The NFAT5-HIF1α-PDGFB love triangle

No great molecular drama is complete without a love triangle. The study presents an interplay between NFAT5, HIF-1α, and platelet-derived growth factor B (PDGFB). Under normal conditions, NFAT5 maintains a delicate balance, preventing excessive PDGFB expression. However, when NFAT5 is removed from the equation, HIF-1α takes the reins and cranks up PDGFB production. The result? Overzealous vascular smooth muscle cells (VSMCs) flood the pulmonary arteries like uninvited guests at a house party, leading to structural remodeling and heightened vascular resistance. This revelation positions NFAT5 as a molecular moderator, ensuring that endothelial responses to hypoxia remain measured rather than catastrophic.

## From bench to bedside, the therapeutic implications

Now that NFAT5 has been thrust into the limelight, the next logical question is: Can one harness its regulatory prowess for therapeutic gain? The potential to mitigate conditions like pulmonary hypertension, notorious for its devastating vascular remodeling and increased right ventricular workload, by targeting the NFAT5-HIF1α-PDGFB axis offers hope in cardiovascular research. Of course, any new therapeutic strategy must first navigate the perilous waters of clinical translation. NFAT5, like any complex regulator, likely has a web of interactions beyond what has been mapped in this study. Tampering with its function could have unintended ripple effects across multiple physiological systems. This study serves as a reminder of the caution and thoroughness required in developing new therapeutic strategies.

## Why this study matters

While it may be tempting to dismiss transcription factors as just another set of molecular cogs in the vast machinery of cellular function, this study reminds one that these proteins are, in many ways, the directors of the biological play. NFAT5 is no exception. By identifying its crucial role in hypoxia-induced endothelial adaptation, the study by Laban and colleagues have expanded the understanding of pulmonary vascular biology and opened up exciting new avenues for therapeutic exploration.

This study also exemplifies the power of multi-omics approaches—integrating transcriptomics, single-cell RNA sequencing, and in vivo models—to unravel the intricacies of cellular adaptation. The depth and breadth of the data presented here provide a robust narrative that could redefine how we view endothelial resilience in the face of hypoxic stress.

## A final thought: the endothelial soap opera continues

As with any good scientific story, this one leaves us with more questions than answers. If NFAT5 is so crucial in endothelial adaptation, what other stress responses might it regulate? Could it have a broader role in systemic vascular homeostasis beyond the pulmonary circulation? And, most tantalizingly, can we manipulate its function to treat pulmonary hypertension and other vascular diseases characterized by maladaptive remodeling?

The stage is set for further exploration, and one thing is clear: NFAT5 is no longer a mere footnote in the transcription factor family when it comes to adaptation of endothelial cells to hypoxia. It has become a key player in the endothelial adaptation saga.

## Data Availability

No datasets were generated or analysed during the current study.
